# Assessing Pretransplant and Posttransplant Therapy Response in Multiple Myeloma Patients

**DOI:** 10.3390/curroncol29110670

**Published:** 2022-11-08

**Authors:** Cristina Potre, Ema Borsi, Ovidiu Potre, Miruna Samfireag, Dan Costachescu, Bianca Cerbu, Felix Bratosin, Cristina Secosan, Rodica Anamaria Negrean

**Affiliations:** 1 Department of Internal Medicine, Discipline of Hematology, “Victor Babes” University of Medicine and Pharmacy, 300041 Timisoara, Romania; 2 Department of Internal Medicine, Discipline of Clinical Practical Skills, “Victor Babes” University of Medicine and Pharmacy, 300041 Timisoara, Romania; 3 Discipline of Radiology and Medical Imaging, “Victor Babes” University of Medicine and Pharmacy, 300041 Timisoara, Romania; 4 Methodological and Infectious Diseases Research Center, Department of Infectious Diseases, “Victor Babes” University of Medicine and Pharmacy, 300041 Timisoara, Romania; 5 Department of Obstetrics and Gynecology, “Victor Babes” University of Medicine and Pharmacy, 300041 Timisoara, Romania; 6 Faculty of Medicine and Pharmacy, University of Oradea, 410073 Oradea, Romania

**Keywords:** multiple myeloma, stem cell transplantation, bortezomib, thalidomide, cyclophosphamide, dexamethasone, lenalidomide

## Abstract

Multiple myeloma (MM) is a hematologic cancer defined by an abnormal development of clonal plasma cells in the bone marrow, releasing vast quantities of immunoglobulins and different proteins. In the majority of patients, MM remains incurable despite decades of medical improvement and a number of treatment breakthroughs. Frontline standard-of-care has little long-term success, with the majority of patients eventually relapsing, although the overall progression-free survival (PFS) has improved significantly in the last ten years. Patients who are eligible for a transplant have the highest PFS rate at 5 years, depending on medication response and other various factors that are yet to be discovered. Therefore, the current study aimed to evaluate the response to VCD (bortezomib, cyclophosphamide, dexamethasone) and VTD (bortezomib, thalidomide, dexamethasone) used as pretransplant regimens, as well as to compare responses between thalidomide and lenalidomide used as maintenance therapy posttransplant. This retrospective study was performed on a group of 105 hospitalized patients in the Hematology Department of the Timisoara Municipal Emergency Clinical Hospital between January 2016 and December 2021. Data was collected from the paper records of patients with MM who were under-followed. The treatment regimens used as induction therapy were either VCD or VTD if cyclophosphamide was contraindicated. Of the 105 patients, 27 became eligible for bone marrow transplantation. Furthermore, they received maintenance therapy which was based on either lenalidomide with dexamethasone or thalidomide with dexamethasone. Of the 62 patients treated with VTD, 17.7% were in complete remission before stem cell transplantation. Of the 43 patients treated with VCD, 37.2% were in complete remission. The 5-year mean progression-free survival (PFS) in the entire cohort was better in the group treated with the VTD regimen (31.6 vs. 27.2 months). However, in the 27 patients undergoing maintenance after ASCT, the PFS with thalidomide was 35.5 months (95% CI = 27–42), while the PFS rate in those receiving maintenance treatment with lenalidomide was 46.1 months (95% CI = 20–73). VCD proved to be superior to VTD in inducing complete pretransplant responses. Regarding maintenance therapy, patients from the lenalidomide group had superior responses compared with those under thalidomide.

## 1. Introduction

Multiple myeloma (MM), the second most prevalent form of hematologic cancer, after leukemias and lymphomas, is characterized by the uncontrolled proliferation of clonal plasma cells [1,2,3]. Researchers have shown that the monoclonal gammopathy of uncertain significance precursor stage is present in almost all instances of multiple myeloma [4]. The secreted plasma cells are hyperproliferative differentiated B-lymphocytes that are capable of secreting a range of immunoglobulins [5]. In most cases, the aberrant plasma cells will proliferate in the bone marrow, and only a small percentage of patients will present with an extramedullary development at the time of diagnosis or acquire extramedullary disease later on in the course of the disease [6,7]. Anemia, renal failure, hypercalcemia, and lytic bone lesions are some of the most common clinical symptoms associated with excessive monoclonal immunoglobulins released from clonal plasma cells, which is the cause of organ damage [8,9].

The overall survival of multiple myeloma has improved significantly in the last ten years, depending on the type and factors of aggressivity [10,11,12]. Patients who are eligible for a transplant have the highest survival rate at five years, which reaches about 80% with modern therapy, in contrast to elderly patients non-eligible for transplantation, whose survival rate is only about 20% at five years [13,14]. However, the prognosis continues to be higher compared with other more aggressive hematologic cancers and much higher than some solid tumors, considerably depending on a variety of prognostic factors [15,16,17]. Despite aggressive therapy that incorporates almost all available drugs and treatment options [18,19,20], strategies to overcome the side effects must be identified in high-risk patients and improve survival in this patient population.

Multiple myeloma is typically sensitive to a variety of cytotoxic drugs, both as an initial treatment and as a treatment for recurrent disease, although the treatments’ effects are transitory, and the MM is not considered curable with current approaches [21]. However, MM treatment has evolved rapidly due to the introduction of new drugs, such as carfilzomib, daratumumab, and pomalidomide [22,23,24]. When deciding on a treatment for multiple myeloma (MM), it is important to take into account a number of criteria that pertain to both the patient and the illness. Age, fragility, and performance status are three of the patient-related characteristics that are considered to be the most relevant. The type of the illness, such as the patient’s risk status, as well as the degree of organ damage, are examples of disease-related parameters [25]. In the case of relapsed or refractory disease, the number, and type of prior treatments, as well as the depth and duration of previous responses, should also be taken into consideration. Therefore, treatment-related factors refer to both the availability of the drug and any adverse events that the drug may cause.

When it comes to the choice of the treatment plan, three-drug regimens are always recommended over two-drug regimens. This is due to the fact that three-drug regimens have repeatedly demonstrated superior responses and survival results compared to two-drug regimens. These regimens include the combination VCD (bortezomib, cyclophosphamide, dexamethasone) and VTD (bortezomib, thalidomide, dexamethasone) [26]. On the other hand, individuals who are already weak may only be able to withstand duplet treatment. Even though multimodal techniques have been used in order to treat multiple myeloma, the most significant obstacle is still the fact that the majority of patients ultimately relapse and become resistant to numerous medication classes [27]. In addition, patients need ongoing treatment throughout the course of the illness, which, due to the possibility of therapy-related side effects, may have a detrimental impact on the quality of life of the patient. It is possible that some patients, due to major comorbidities, will not be candidates for certain systemic medicines or autologous transplantation. This might drastically restrict the pool of viable modalities that can be deployed in the fight against their illness. In addition, despite the recent incorporation of a number of novel agents into standard clinical practice, there are still no reliable markers that can accurately predict a patient’s reaction to a particular drug class, which severely limits the capacity to choose a more individualized approach to therapeutic intervention [28]. Therefore, this paper aims to evaluate the response to the two treatment protocols used as pretransplant treatment, to compare the evolution of patients to posttransplant therapy and their survival rate.

## 2. Materials and Methods

### 2.1. Study Design and Settings

Patients were enrolled in the current observational retrospective study if their hospital admission occurred between January 2016 and December 2021. The research was carried out at the “Victor Babes” University of Medicine and Pharmacy from Timisoara in the Hematology Department of the Timisoara Municipal Emergency Clinical Hospital. As a retrospective study, data was collected from the paper records and digital records of patients diagnosed in that period with multiple myeloma and followed up in evolution.

The hematology clinic affiliated with the “Victor Babes” University of Medicine and Pharmacy, as an auxiliary of the Municipal Clinical Emergency Hospital from Timisoara, operates under the laws of the local commission of ethics that approves scientific research that functions in accordance with the International Conference on Harmonization from Helsinki regarding technical requirements for registration of pharmaceuticals for human use. The research complied with the ethics criteria from the university where the study was developed and was approved by the ethics committee of both institutions.

### 2.2. Participants and Protocols

**Figure 1:**
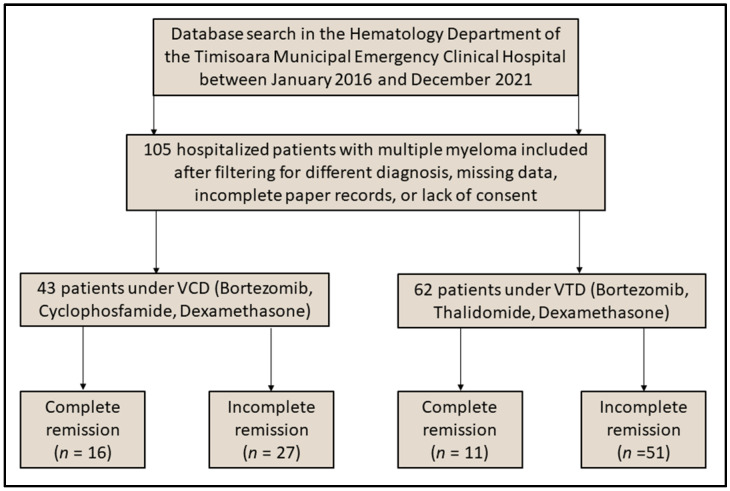
Flowchart of patients considered for inclusion in the study.

Adult patients with a history of multiple myeloma were considered eligible for inclusion in the current study, identified by the International Classification of Diseases (ICD-10) diagnosis codes [29]. The main inclusion criteria for these were considered as the history of treatment with the VCD scheme (Bortezomib, Cyclophosphamide, Dexamethasone) and VTD scheme (Bortezomib, Thalidomide, Dexamethasone). Other criteria comprised the patient age under 70 years, the evidence of immune-electrophoresis showing no monoclonal immunoglobulins, the patients should not have major comorbidities that might bias survivability, no severe pulmonary restriction or obstruction, no evidence of heart failure (ejection fraction > 50%), chronic kidney failure (eGFR < 60 mL/min per 1.73 square meters), and other types of malignancies. Patients were removed from the research if their medical records were found to be lacking important data or if the permission form was not completed in the existing documents, as shown in Figure 1. It was determined using a convenience sampling method that a total of 33 patients would be sufficient for inclusion in each group for the results to have statistical power at a 99% confidence level and a 1% lifetime risk of developing MM [30].

The treatment protocol used in our clinic as first-line pretransplant therapy was based either on VCD or VTD, depending on renal function. The regimen of choice was VCD, but in patients with kidney failure, thalidomide was administered instead of cyclophosphamide, considering a lower safety profile compared with thalidomide. Also, cyclophosphamide was used in patients with a GFR higher than 30 mL/min, and was avoided in patients presenting hematuria, as well as in those presenting increased BUN levels. After ASCT, patients with reduced kidney function received granulocyte growth-stimulating factor (G-CSF) and adjusted doses of melphalan. The VCD protocol includes: (1) bortezomib 1.3 mg/m^2^ body surface area (BSA) on days 1, 8, 15, and 22; (2) cyclophosphamide 500 mg/m^2^ BSA on days 1, 8, and 15; and (3) dexamethasone 20 mg/day on days 1–2, 4–5, 8–9 and 11–12. The treatment is repeated every 21 days and is administered in 6–8 cycles. The VTD regimen protocol was as follows: (1) bortezomib 1.3 mg/m^2^ BSA on days 1, 4, 8, and 11; (2) thalidomide 100 mg/day, orally, administered continuously; and (3) dexamethasone 40 mg/day on days 1–2, 4–5, 8–9, and 11–12. The treatment is repeated every 21 days and was administered in 6–8 cycles as well. Concerning the maintenance treatment, it was based on either administration of lenalidomide 25 mg once daily for three weeks and dexamethasone 40 mg once a week for three weeks, which was repeated every 28 days, or thalidomide 100 mg continuously and dexamethasone 40 mg once a week continuously. These two maintenance treatments were chosen to preserve kidney function by avoiding renal toxicity.

### 2.3. Variables

The variables of interest for the current study comprised: (1) background characteristics—age, sex, body mass index (BMI), area of residence, relationship status, occupation, substance use behavior, chronic comorbidities; (2) multiple myeloma characteristics—multiple myeloma staging according to the Salmon-Durie staging system [31], translocations, chromosomal abnormalities, GEP70 risk assessment, cytogenic risk assessment, Karnofsky performance scale, myeloma type, urine immunofixation assessment; (3) laboratory parameters pretreatment and posttreatment; (4) complications and outcomes (drug-related and disease-related); and (5) follow-up results in patients who received autologous stem cell transplant.

### 2.4. Statistical Analysis

IBM SPSS version 27.0 (SPSS. Inc., Chicago, IL, USA) and Microsoft Excel (Microsoft Corp. Redmond, WA, USA) were the software used for statistical analysis. The representation of categorical variables was accomplished by absolute values and the frequencies of those values. A statistical examination of the proportions was carried out with chi^2^ and Fisher’s exact tests. A Shapiro-Wilk test was performed to determine the Gaussian distribution of data, and a Student’s *t*-test was carried out to compare the means of Gaussian variables. The Wilcoxon test was used for nonparametric, non-normally distributed variables. The Kaplan-Meyer curve was used to estimate survival. A level of significance of 0.05 was chosen as the threshold for the alpha value.

## 3. Results

### 3.1. Patient Characteristics

Table 1 describes the comparison of the study cohort background characteristics before pretransplant therapy. There were a total of 105 study participants, 62 of them being treated with the VTD scheme, and the other 43 with the VCD scheme. Of the 105 patients included in the study, 42 (40.0%) fell into the age group of 65–75 years, while the majority of 47 patients were older than 75 years. It was observed that 67 patients were men (63.8%), and more than 55% were residing in an urban environment. A total of 35.5% of patients in the VTD group had a BMI higher than 25, and a similar number of patients in the VCD group were overweight and obese (39.5%). There were no statistically significant differences between study groups regarding their relationship status, occupation, substance use behavior, and chronic comorbidities. The most common comorbid condition was high blood pressure in more than 55% of all patient cohorts.

### 3.2. Multiple Myeloma Characteristics

Table 2 describes the multiple myeloma characteristics in the two study groups before pretransplant therapy. According to the Salmon-Durie staging system, patients with multiple myeloma in this study were mostly stage three (72.6% in the VTD group, respectively 55.8% in the VCD group), although the difference was not statistically significant. The Karnofsky performance status was over 90 in almost two-thirds of all patients, while one-third had high cytogenic risk. The most common myeloma type was IgG type in 56.3% of the VTD patients and 47.7% in the VCD group). Positive immunofixation was identified in approximately 55% of the entire cohort group.

The description of laboratory findings before and after pretransplant therapy is presented in Table 3. Among the significant findings in the VTD group, it was observed that total protein count was statistically lower after the VTD treatment scheme ended (from 62.9% of patients outside the normal range to 45.2%, *p*-value = 0.047). Also, creatinine levels improved, as well as the WBC that normalized after treatment. However, the BUN levels were higher after treatment, and the liver enzymes were significantly more elevated in the VTD posttreatment group (from 33.9% to 53.2%, *p*-value = 0.029).

Regarding the VCD cohort, it was also observed that total proteins decreased after treatment, as well as a significant decrease in calcium levels (51.2% values outside the normal range vs. 30.2% after treatment, *p*-value = 0.048), and a normalization of WBC. Similarly to the VTD group, the liver enzymes were significantly more elevated (30.2% vs. 53.5%, *p*-value = 0.028).

**Figure 2:**
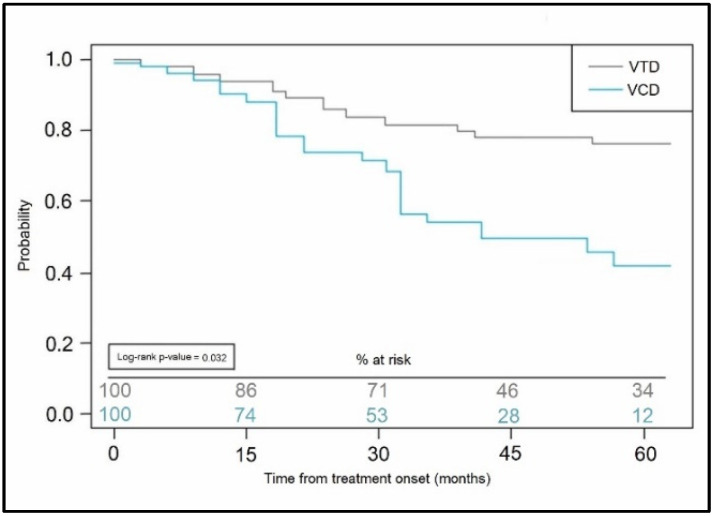
Kaplan-Meyer probability curve of progression-free survival stratified by induction treatment scheme.

The patients included in the current study were also analyzed in follow-up to determine any significant differences between the drug-related complications, disease-related complications, and treatment outcomes, such as treatment response and progression-free survival. Neutropenia was more common as a drug complication in the VCD group (34.9% vs. 17.7%, *p*-value = 0.045), followed by kidney damage (25.6% vs. 8.1%, *p*-value = 0.014), and respectively anemia (22.7% in the VCD group vs. 6.5% in the VTD group, *p*-value = 0.012), as presented in Table 4. Complete response was achieved significantly more in patients treated with VCD; however, the VTD group encountered fewer ICU admissions and better progression-free survival, as seen in Figure 2. Although the progression-free survival was significantly better in the VTD group, the PFS after maintenance with thalidomide (*n* = 11) was lower than in those undergoing maintenance with lenalidomide (*n* = 16).

## 4. Discussion

### 4.1. Literature Findings

The prognosis for individuals with multiple myeloma has greatly improved over the last 20 years because of developments in therapy, particularly the introduction of innovative medicines that are now considered to be the standard of care. The results may be affected by a variety of circumstances, including the pretransplant response and the cytogenetic risk. It has been shown that attaining a full response or a very excellent partial response is essential for assuring a prolonged progression-free survival or overall survival.

According to the findings of previous research, the pre-and post-autologous stem cell transplant response grew significantly from 5% to 50% in the first trials and then respectively from 30% to almost 80% [10]. The significance of being in remission prior to transplantation is still a matter of debate. According to the findings of research conducted by the Korean myeloma group, having a full response before an autologous stem cell transplant was related to a longer overall survival time after the transplant.

Several studies have shown that in transplantation, the use of conventional chemotherapy approaches, such as VTD followed by autologous stem cell transplant, is associated with poor outcomes in high-risk patients when compared with novel therapies [32,33]. However, when adding a new molecule, such as the monoclonal antibody antiCD38, daratumumab, the situation changes drastically. This may be seen in several studies, such as Cassiopeia [34]. There are many alternatives when it comes to myeloma treatment, but risk assessment is very important before choosing a certain regimen [35]. VCD and VTD continue to be used as induction regimens before transplant, and they still induce a very good response in the majority of the patients. VTD proved to be superior to VCD in a prospective trial published in 2016. That was mainly due to less hematologic toxicity in the VTD arm [36]. Similarly, in our study, it can be concluded that patients in the VTD arm had better outcomes, which can be attributed to the fact that some of the patients undergoing VCD have developed significant cytopenia and associated severe infections. On the other hand, when it came to the VTD treatment scheme, there were also some patients who developed cytopenia, but the majority were not at the same degree of severity and required no further dose changes. Consequently, there were very few patients who developed severe neutropenia and secondary infections, therefore resulting in fewer ICU admissions.

On the other hand, different studies report daratumumab-refractory multiple myeloma and the role of salvage autologous stem cell transplantation in these patients. The use of a salvage autologous stem cell transplant in patients who have universal exposures to numerous new drugs and refractoriness to daratumumab is something that has been debated recently, and it has even been demonstrated that high-dose chemotherapy followed by autologous stem cell transplant may generate considerable responses in 80% of patients, with an estimated 25% of these patients anticipated to be alive at 36 months after treatment [37]. It would seem that the response rates and survival outcomes of salvage autologous stem cell transplant are similar to recent findings with new Car-T cell therapy [38], which suggests that salvage autologous stem cell transplant represents a legitimate option in some individuals. Despite the fact that progression-free survival and overall survival in this cohort may have been influenced by maintenance post-salvage autologous stem cell transplant, the impact of the regimens used in maintenance has been shown to be rather small in this refractory patient population, with short progression-free survival and overall survival [39]. This highlights the fact that salvage autologous stem cell transplant contributes majorly to the improved outcome.

Other newer treatment schemes for induction for multiple myeloma with bortezomib include the anti-SLAMF7 monoclonal antibodies, such as elotuzumab. Elotuzumab was shown to be more effective when paired with standard antimyeloma drugs, such as lenalidomide and bortezomib, according to preliminary experimental investigations. This was later verified in clinical studies, which showed that elotuzumab had a moderate amount of activity when used alone, but a much higher amount of activity when paired with either lenalidomide or pomalidomide. Elotuzumab is now licensed for the treatment of relapsed or refractory illness when combined with either pomalidomide/dexamethasone or lenalidomide/dexamethasone. In addition, elotuzumab has been investigated in conjunction with bortezomib, with promising findings from those studies. A phase 3 study that is currently being conducted and is showing encouraging first findings is investigating the potential benefits of adding elotuzumab to the triplet combination of arfilzomib/lenalidomide/dexamethasone for use in the upfront and posttransplant maintenance settings [40,41].

When it comes to posttransplant maintenance treatment, there are several studies that prove the efficiency of lenalidomide [42]. Actually, lenalidomide became the standard of care after autologous transplant as it proved to extend survival and delay relapse [43,44]. Use of thalidomide as maintenance therapy is very controversial. There are several studies that showed that the use of thalidomide as a maintenance therapy after autologous stem cell transplantation had been associated with improved progression-free survival, but no significant improvement in overall survival was detected [45,46].

Concerns have been raised regarding the ability of elderly individuals to withstand the physically taxing treatment regimen required for multiple myeloma illnesses. The population as a whole is becoming older. Because of the increased risk of toxicity associated with treating older individuals, less strenuous treatment regimens are often given priority. However, since the median age at which multiple myeloma is diagnosed is 69 years, establishing an age threshold for transplant would result in the elimination of a sizable group of individuals [10]. According to the findings of studies, there is a general upward tendency in the number of patients, which ranges from 0% in the group of young patients to 25% in those who were older than 65 years of age when they had their transplant [47]. It was discovered that there was a statistically significant increase in progression-free survival as well as overall survival for patient groups ranging in age from under 65 to over 65 years old. In our study, the posttransplant survival rate was 92.7%, with no statistically significant differences between the two treatment lines, and the mean survival in patients treated with thalidomide was 35 months (95% CI = 27–42), while the mean survival rate in those who underwent maintenance treatment with lenalidomide was 46 months (95% CI = 20–73).

### 4.2. Study Limitations and Future Perspectives

The current study successfully met the participants’ inclusion criteria and sample size requirements for statistical significance. Nevertheless, several limitations exist. First, the retrospective study design impacts our results as the research depends on the accuracy of both patient information tracking and the digital transcription of data from paper records. Also, different biasing factors can occur during patient selection, treatment selections, as well as during the follow-up period. Although previous results are sometimes contradictory, the maintenance treatment with thalidomide was considered in the current study due to medication availability in Romania, thalidomide’s good kidney safety record, and its ease of use, as patients do not need to be hospitalized when receiving maintenance, unlike bortezomib, and it does not require very frequent monitoring. Although our exclusion criteria tried to identify any confounding factors for disease progression and survival, the retrospective design makes it impossible to exclude them all. It should also be mentioned that the study population is relatively homogenous since there is a low population diversity in the country of study. Therefore, different results at follow-up might occur when compared to studies performed in other parts of the world. Nevertheless, further studies are required to investigate the benefits of using thalidomide and lenalidomide as maintenance treatments on large cohorts of patients and for a longer duration to help establish clear guidelines for disease maintenance.

## 5. Conclusions

In conclusion, the unfavorable prognosis is likely to materialize as a result of a diverse assortment of underlying causes. It is necessary to devise methods for overcoming the effects of the unfavorable prognosis, and it is also crucial to investigate the mechanisms by which each variable contributes to the formation of the unfavorable prognosis. Both thalidomide and cyclophosphamide in combination with bortezomib and dexamethasone are indicated as induction therapy pretransplant in multiple myeloma; however, lenalidomide used as maintenance therapy has been associated with a better survival rate in post-transplant patients when compared to thalidomide. Additional research is required to determine the optimum length of time for maintenance treatment.

## Figures and Tables

**Table 1:** Comparison of the study cohort background characteristics before pretransplant therapy.

Variables	VTD (*n* = 62)	VCD (*n* = 43)	*p*-Value *
Age			0.935
18–64 years	10 (16.1%)	6 (14.0%)	
65–75 years	25 (40.3%)	17 (39.5%)	
>75 years	27 (43.5%)	20 (46.5%)	
Sex			0.816
Men	39 (62.9%)	28 (65.1%)	
Women	23 (37.1%)	15 (34.9%)	
BMI			0.107
Underweight (<18.5 kg/m^2^)	8 (12.9%)	6 (14.0%)	
Normal weight (18.5–25.0 kg/m^2^)	32 (51.6%)	20 (46.5%)	
Overweight (>25.0 kg/m^2^)	22 (35.5%)	17 (39.5%)	
Area of residence (urban)	34 (54.8%)	26 (60.5%)	0.566
Relationship status (married)	57 (91.9%)	40 (93.0%)	0.836
Occupation (retired)	52 (83.9%)	37 (86.0%)	0.760
Substance use behavior			
Frequent alcohol consumption	11 (17.7%)	9 (20.9%)	0.682
Frequent smoker	20 (32.3%)	16 (37.2%)	0.599
Chronic comorbidities **			
High blood pressure	38 (61.3%)	24 (55.8%)	0.574
Lung	10 (16.1%)	6 (14.0%)	0.760
Metabolic	13 (21.0%)	9 (20.9%)	0.996
Cerebrovascular	16 (25.8%)	15 (34.9%)	0.315
Digestive & liver	6 (9.7%)	4 (9.3%)	0.948
Depression	7 (11.3%)	4 (9.3%)	0.743
Other	5 (8.1%)	3 (7.0%)	0.836

* Chi-squared or Fisher’s exact test. ** Excluding severe pulmonary restriction or obstruction, evidence of heart failure, kidney failure, and other types of malignancies. BMI—body mass index.

**Table 2:** Multiple myeloma characteristics in the two study groups before pretransplant therapy.

Variables	VTD (*n* = 62)	VCD (*n* = 43)	*p*-Value *
Salmon-Durie staging			0.124
1	5 (8.1%)	3 (7.0%)	
2	12 (19.4%)	16 (37.2%)	
3	45 (72.6%)	24 (55.8%)	
Translocation t(4;14)	9 (14.5%)	7 (16.3%)	0.804
Chromosome 17p deletion	5 (8.1%)	5 (11.6%)	0.540
High GEP 70 risk score at diagnosis	18 (29.0%)	14 (32.6%)	0.699
High cytogenic risk	17 (27.4%)	12 (27.9%)	0.956
Karnosfky performance status ≥ 90	34 (54.8%)	26 (60.5%)	0.566
Myeloma type			0.887
IgA	13 (20.3%)	10 (22.7%)	
IgG	36 (56.3%)	21 (47.7%)	
IgD	4 (6.3%)	4 (9.1%)	
Light Chain	7 (10.9%)	4 (9.1%)	
Nonsecretory	2 (3.1%)	2 (4.5%)	
Others	2 (3.1%)	3 (6.8%)	
Positive urine immunofixation	34 (54.8%)	25 (58.1%)	0.737

* Chi-squared or Fisher’s exact test; GEP70—gene expression profiling.

**Table 3:** Laboratory findings before and after pretransplant therapy.

Variables	VTD (*n* = 62)		*p*-Value *	VCD (*n* = 43)		*p*-Value *
% Outside Normal Range	Pretreatment	Posttreatment		Pretreatment	Posttreatment	
Albumin, g/L	22 (35.5%)	20 (32.3%)	0.704	14 (32.6%)	11 (25.6%)	0.476
Total proteins	39 (62.9%)	28 (45.2%)	0.047	30 (69.8%)	21 (48.8%)	0.048
Creatinine level, mmol/L	32 (51.6%)	17 (27.4%)	0.005	12 (27.9%)	16 (37.2%)	0.357
BUN (mmol/L)	14 (22.6%)	28 (45.2%)	0.007	9 (20.9%)	16 (37.2%)	0.096
GFR	7 (11.3%)	13 (21.0%)	0.142	11 (25.6%)	17 (39.5%)	0.167
Hemoglobin level, g/dL	18 (29.0%)	16 (25.8%)	0.687	9 (20.9%)	17 (39.5%)	0.060
Calcium level, mmol/L	29 (46.8%)	25 (40.3%)	0.468	22 (51.2%)	13 (30.2%)	0.048
RBC (millions/mm^3^)	16 (25.8%)	15 (24.2%)	0.835	10 (23.3%)	9 (20.9%)	0.794
PLT (thousands/mm^3^)	17 (27.4%)	13 (21.0%)	0.401	10 (23.3%)	8 (18.6%)	0.596
WBC (thousands/mm^3^)	37 (59.7%)	24 (38.7%)	0.019	28 (65.1%)	18 (41.9%)	0.030
ALT (U/L)	21 (33.9%)	33 (53.2%)	0.029	13 (30.2%)	23 (53.5%)	0.028
AST (U/L)	19 (30.6%)	30 (48.4%)	0.043	14 (32.6%)	23 (53.5%)	0.049

* Chi-squared or Fisher’s exact test; RBC—red blood cells; PLT—platelets; WBC—white blood cells; BUN—blood urea nitrogen; GFR—glomerular filtration rate.

**Table 4:** Complications and outcomes during pretransplant therapy.

Variables	VTD (*n* = 62)	VCD (*n* = 43)	*p*-Value *
Drug complications			
Neutropenia	11 (17.7%)	15 (34.9%)	0.045
Thrombocytopenia	3 (4.8%)	5 (11.6%)	0.197
Anemia	4 (6.5%)	10 (22.7%)	0.012
Pancytopenia	4 (6.5%)	4 (9.3%)	0.588
Neuropathy	11 (17.7%)	3 (7.0%)	0.110
Kidney damage	5 (8.1%)	11 (25.6%)	0.014
Elevated liver enzymes	13 (21.0%)	10 (22.7%)	0.780
Others	5 (8.1%)	5 (11.6%)	0.540
Disease-related complications			
Infection	18 (29.0%)	11 (25.6%)	0.697
Fractures	4 (6.5%)	2 (4.7%)	0.695
Anemia	18 (29.0%)	13 (30.2%)	0.894
Reduced kidney function	32 (51.6%)	12 (27.9%)	0.015
Others	5 (8.1%)	2 (4.7%)	0.490
Treatment response			0.033
CR	11 (17.7%)	16 (37.2%)	
PR	12 (19.4%)	9 (20.9%)	
SD	12 (19.4%)	10 (23.3%)	
PS	27 (43.5%)	8 (18.6%)	
ICU admissions	4 (6.5%)	9 (20.9%)	0.026
Death	3 (4.8%)	4 (9.3%)	0.367
Mean progression-free survival, (months)	31.6 ± 9.9	27.2 ± 10.4	0.030
Maintenance after ASCT	Thalidomide (*n* = 11)	Lenalidomide (*n* = 16)	
Mean progression-free survival, (months)	35.5 ± 10.3	46.1 ± 11.8	0.023

* Chi-squared or Fisher’s exact test; ICU—intensive care unit; CR—complete response; PR—partial response; SD—stable disease; PS—progression; ASCT—autologous stem cell transplant.

## Data Availability

The data presented in this study are available on request from the corresponding author.
